# Prenatal Imaging of Micrognathia, Micromelia, and Fetal Hydrops Leading to the Diagnosis of Achondrogenesis Type II with a *COL2A1* Missense Mutation

**DOI:** 10.3390/ijms262311472

**Published:** 2025-11-27

**Authors:** Yi-Cheng Wu, Chih-Yao Chen, Guan-Yeu Chen, Ching-Hua Hsiao, Woei-Chyn Chu, Jack Yu-Jen Huang

**Affiliations:** 1 Department of Biomedical Engineering, National Yang-Ming Chiao-Tung University, Taipei 112304, Taiwan; dam86@tpech.gov.tw (C.-H.H.); wchu@nycu.edu.tw (W.-C.C.); 2 Department of Obstetrics and Gynecology, Ton Yen General Hospital, Hsinchu 302048, Taiwan; jyjhuang@stanford.edu; 3 Taiwan IVF Group Center for Reproductive Medicine & Infertility, Hsinchu 302053, Taiwan; 4 Dianthus MFM Center Minquan, Dianthus MFM Group, Taipei 114067, Taiwan; zachary.joyce@icloud.com; 5 Department of Obstetrics and Gynecology, Taipei Veterans General Hospital, Taipei 112201, Taiwan; gychen@vghtpe.gov.tw; 6 Department of Obstetrics and Gynecology, Fuyou Branch, Taipei City Hospital, Taipei 100027, Taiwan

**Keywords:** type II collagen disorders, achondrogenesis type II, missense mutation, hydrops, *COL2A1*

## Abstract

This case report describes a fetus with achondrogenesis type II, a severe and lethal type II collagen disorder, presenting with micrognathia and hydrops. Prenatal evaluation with 2D/3D ultrasound, followed by postmortem imaging and pathological examination, confirmed the diagnosis. Genetic testing revealed a heterozygous *COL2A1* mutation (1703G>A; Gly516Ser, exon 24). The significance of this study lies in the identification of a missense mutation in *COL2A1* associated with achondrogenesis type II. This report highlights that the condition may present with hydrops and craniofacial anomalies, establishing this variant as a pathogenic mutation associated with the disorder.

## 1. Introduction

Achondrogenesis disorder is one of the lethal short-limb dwarfisms. Type I (Parenti–Fraccaro type) of achondrogenesis (OMIM #200600) is more severe and characterized by severe micromelia, multiple rib fractures, and lack of ossification of the calvarium and spine [1,2]. Type II (Langer–Saldino type) achondrogenesis (OMIM #200610) is less severe and characterized by the absence of rib fractures, varied calcification of the calvarium and spine, more variable limb shortening, and heavier birth weight [3,4]. Achondrogenesis type II (OMIM #200600) is caused by highly disruptive pathogenic variants in the *COL2A1* gene, leading to nearly complete failure of cartilage formation and a lethal skeletal phenotype. The *COL2A1* gene is located on chromosome 12q13.11, encodes the alpha one chain, and is expressed primarily in cartilage [5]. Abnormalities in the *COL2A1* gene can induce severe heritable diseases, such as achondrogenesis type II, hypochondrogenesis (OMIM #200610), platyspondylic dysplasia (OMIM #151210) [6,7,8], spondyloepiphyseal dysplasia (SED) (OMIM #183900, OMIM #156550) [9,10,11], spondyloepiphyseal dysplasia congenita (SEDC) (OMIM #183900) [12,13], Kniest dysplasia (OMIM #156550) [14,15,16], spondyloperipheral dysplasia (OMIM #271700) [17,18,19], and Stickler syndrome (OMIM #108300) [20,21,22,23,24]. Here, we present a case of achondrogenesis type II, characterized by micrognathia, micromelia, and hydrops fetalis. In utero three-dimensional ultrasound, postmortem X-ray, and pathologic report are also demonstrated.

## 2. Case Presentation

**Figure 1:**
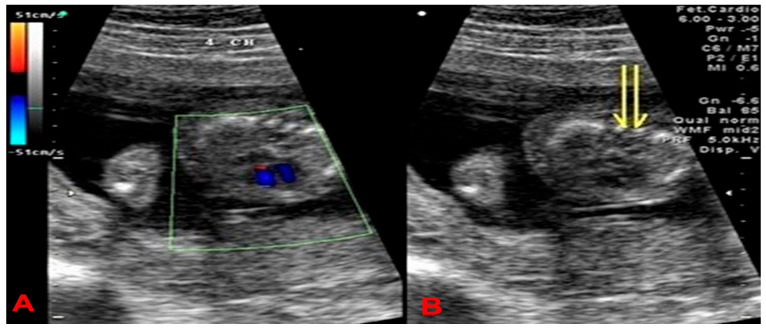
A transverse view of the fetal thorax showed a small chest, and a normal four-chamber view under color Doppler ultrasound (**A**) and absence of ossification of the vertebral body (two yellow arrows indicate only ossification of vertebral pedicles in the right figure, (**B**)).

**Figure 2:**
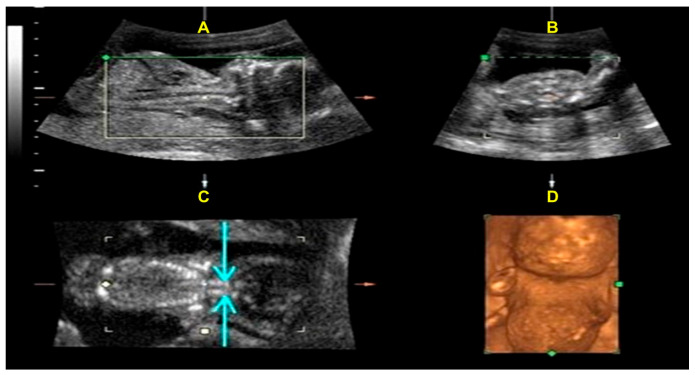
The coronal view of the fetal neck on 3D ultrasound showed absent ossification of the vertebral bodies (the light blue arrows in the left lower figure indicate only ossification of vertebral pedicles) (**C**); the 3D ultrasound revealed a flat face, micrognathia (**A**,**D**), and a small and narrow chest (**B**,**D**).

A 27-year-old woman, gravida 1 para 0, was referred to our hospital at 18 weeks of gestation due to abnormal sonographic findings of hydrops and shortening of limbs. The parents denied genetically related anomalies. There was no family history of congenital malformations. The tertiary level ultrasound examination (GE Voluson 730; RAB4-8 transducer) revealed a single fetus with a biparietal diameter of 4.13 cm (73%), a frontal-occipital diameter of 5.18 cm (81.4%), a head circumference of 14.98 cm (42.7%), normal skull ossification, an abdominal circumference of 12.23 cm (43.0%), a femur length of 1.18 cm (<1%), a humerus length of 1.37 cm (<1%), an ulnar length of 1.16 cm (<1%), a radius length of 1.13 cm (<3%), a tibia length of 1.22 cm (<1%), a fibula length of 1.03 cm (<1%) [Supplementary Materials of long bones]. The thoracic-to-abdominal circumference ratio is 0.920 (>0.89). The transverse view of the fetal thorax revealed a small chest and an absence of ossification of the vertebral body (Figure 1). Other notable findings included obvious skin edema (4.5 mm) and a nuchal thickness of 8.96 mm. The 3D ultrasound study revealed absent vertebral body ossification in the fetal neck coronal view; moreover, a flat face, micrognathia, micromelia, and a small, narrow chest are noteworthy findings (Figure 2). The chromosome karyotype was 46, XX, as determined by amniocentesis.

## 3. Materials and Methods

### Molecular Study

Mutation analysis in the *COL2A1* gene:

Fresh fetal lower leg skin tissues were obtained from surgically resected specimens. Proteinase K digestion of the tissue was carried out in a volume of 2 mL tissue lysis buffer containing 0.2 M Tris-HCl, pH 8.0, 100 mM EDTA, 1% SDS, and proteinase K (1 mg/mL final concentration) at 370 °C overnight. DNA was purified by extracting it with phenol-chloroform-isoamyl alcohol (25:24:1), followed by precipitation with isopropanol. The precipitate was washed with 70% ethanol and dissolved in an appropriate volume of TE to determine DNA concentration.

The promoter, 54 exons, and the flanking intronic sequences of the *COL2A1* gene were amplified using 39 primer pairs designed with Primer3. PCR amplifications were performed in a 10 μL volume containing 25 ng or 50 ng of genomic DNA, 0.5 U AmpliTaq DNA polymerase (Applied Biosystems, Foster City, CA, USA), two pmol of each primer, 250 μmol/L of each dNTP, 2 mmol/L MgCl_2_, and one μL 10× reaction buffer. The PCR conditions consisted of an initial 5-min incubation at 95 °C, followed by 45 cycles of 95 °C for 30 s, an annealing temperature for 30 s, and 72 °C for 45 s, followed by a 5-min incubation at 72 °C. The resulting PCR fragments were purified using 2 U exonuclease I and 1 U shrimp alkaline phosphatase. For direct sequencing, single-stranded DNA fragments were generated in a second round of amplification using a purified aliquot (2 μL) from the first PCR and forward or reverse primer (3.2 pmol). Sequencing reaction products were electrophoresed on an ABI PRISM 3730 DNA sequencer (Applied Biosystems, Foster City, CA, USA). The results were analyzed using the Phred/Phrap/Consed pipeline (http://www.phrap.org/) and the PolyPhred software (version 10) [25].

## 4. Results

**Figure 3:**
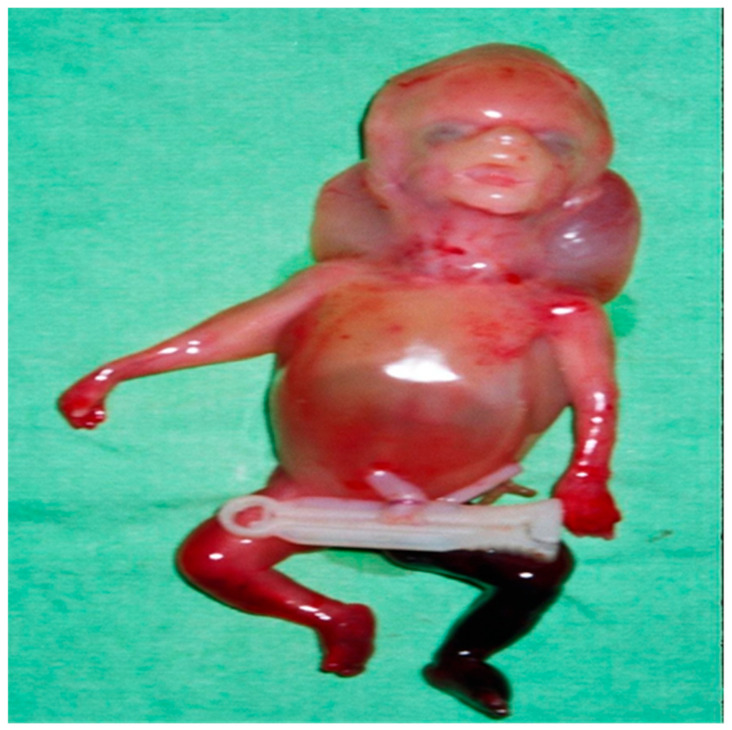
A stillborn female, weighing 200 g, was delivered after vaginal induction.

**Figure 4:**
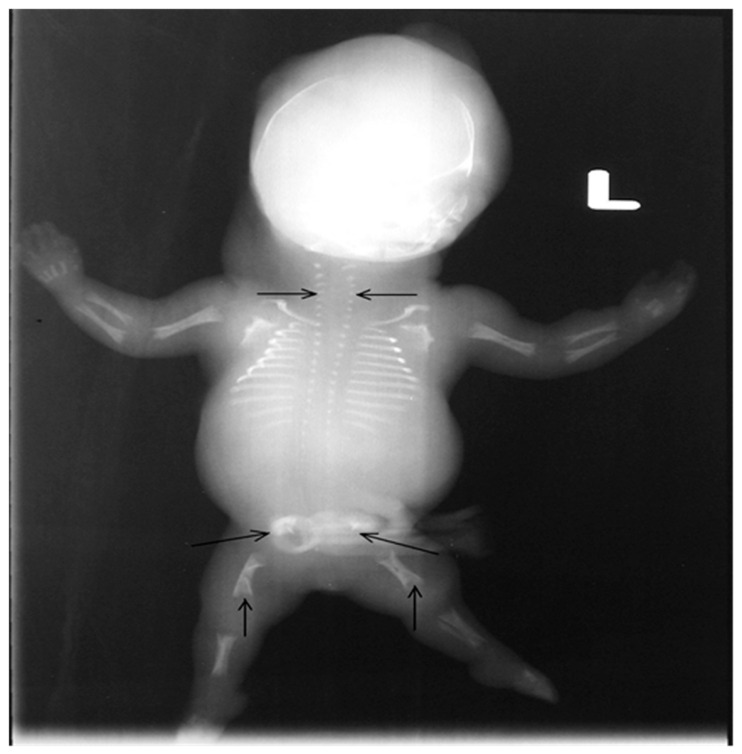
The post-mortem radiograph showed a normal ossified skull, short ribs, only ossified vertebral pedicles of the spine (horizontal arrow), short, long bones, hypoplastic ilium with a medial spike, and hypoplastic ischium (oblique arrow). The tubular bones were short and broad with flared and cupped metaphyseal margins (vertical arrow).

**Figure 5:**
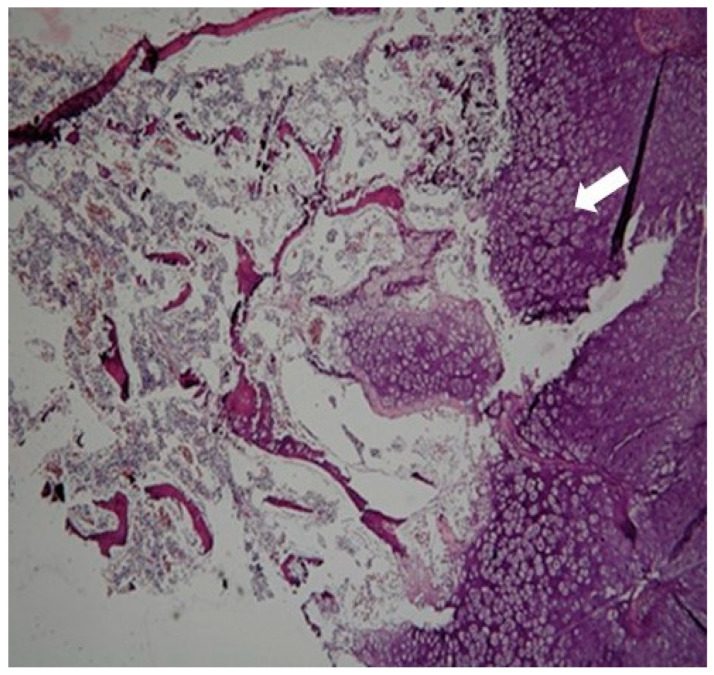
The pathological finding revealed immature cartilage tissue with prominent vascular channels and abundant perivascular fibrosis at the osteochondral junctions of the limbs (white arrow). In addition, the disorganization of the growth plate and irregularity between cartilage columns and bone trabeculae were distinctive.

After consulting with their parents, the termination of the pregnancy was decided by them. A stillborn female, weighing 200 g, was delivered after vaginal induction at 19 weeks of gestation (Figure 3). The post-mortem radiograph showed a normal ossified skull, short rib, only ossified post pedicles of the spine, short, long bones, hypoplastic ilium with medial spike, and hypoplastic ischium (Figure 4). Tissue samples from the fetus were fixed, dehydrated, embedded in paraffin, sectioned, and subjected to hematoxylin–eosin (H&E) staining following standard protocols for histological analysis. The pathological finding revealed immature cartilage tissue with prominent vascular channels and abundant perivascular fibrosis at the osteochondral junctions of the limbs. In addition, the disorganization of the growth plate and irregularity between cartilage columns and bone trabeculae were distinctive. A few foci of spindle-shaped fibroblast-like chondrocytes were identified (Figure 5). The lungs showed hypoplasia, and the neck soft tissue showed congestion and edema. No inflammation or infarction was seen.

**Figure 6:**
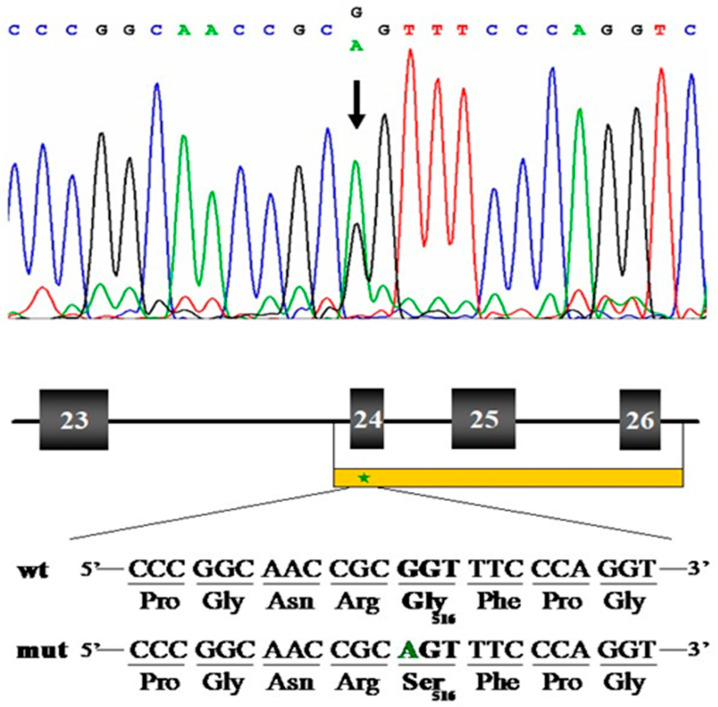
Nucleotide sequence analysis of exon 24, 25, and 26 portions of *COL2A1* and adjacent intronic regions of the affected individual. The individual was heterozygous for the transition, indicated by the arrow. The approximate location for PCR amplification was marked by a yellow bar beneath the schematic, a partial drawing of the *COL2A1* genomic structure. A green asterisk indicated the mutation site. The translated products were shown below the normal and mutant sequences, respectively.

After delivery, fresh fetal lower leg skin tissue was obtained from surgically resected specimens. Molecular analysis [Figure 6] was performed on genomic DNA; the report revealed heterozygosity for a 1703G>A transition (GenBank accession number NM_001844), resulting in a glycine to serine substitution at amino acid position 516 (GenBank accession number NP_001835) in the COL2A1 protein [Supplementary Materials Table S1 and Supplementary S1]. To determine whether this mutation is new, we compared it against known databases of mutations, including ClinVar (a database of human genetic variants), HGMD (Human Gene Mutation Database), and dbSNP (a database of single-nucleotide polymorphisms).

## 5. Discussion

Achondrogenesis Type II (ACG 2; OMIM #200610) is a severe skeletal disorder caused by dominant mutations in the *COL2A1* gene, which encodes type II collagen [26,27]. These mutations typically occur within the gene’s triple-helical region, with glycine substitutions being the most common, accounting for approximately 94% of cases [26]. Characteristic features of ACG 2 include severe micromelic dwarfism, a small chest, a prominent abdomen, incomplete ossification of the vertebral bodies, and disorganization of the costochondral junction. The condition is often lethal, with death usually occurring either in utero or in the early neonatal period [28].

The first case of ACG 2 was described in 1989 and was associated with jugular lymphatic obstruction [29]. The clinical presentation may include increased nuchal thickness, noticeable skin edema, and hydrops fetalis, which can result from obstruction of lymphatic flow. Prenatal ultrasound typically demonstrates severe micromelia, normal to slightly reduced skull ossification, absence of vertebral ossification, and short ribs without fractures. The most frequent mutation reported is a glycine substitution at position 1110 (p.Gly1110Cys, c.3328G>T), identified in approximately 11% of families [26]. While glycine substitutions are the most common mutations, they highlight the crucial role of glycine residues in maintaining the structural integrity of type II collagen. By contrast, hypochondrogenesis is a milder form of skeletal dysplasia that shares several features with ACG 2 but presents with less severe manifestations. Both conditions are fatal in the perinatal period. Based on the cited publications, approximately 50 cases had been reported as of 2020, with a higher prevalence in females. The average age at diagnosis for ACG 2 is 0.54 years, which is notably young for a type II collagenopathy [30].

The pathogenesis of ACG 2, caused by missense mutations in the *COL2A1* gene, involves two mechanisms.

A. The substitution of a single amino acid in the collagen protein sequence disrupts the structure and function of the collagen triple helix. This mutation often results in the production of abnormal collagen molecules that cannot form the proper fibrils required for cartilage function. These defective collagen molecules are prone to misfolding or aggregation, leading to rapid degradation or accumulation in the endoplasmic reticulum (ER) and causing cellular stress. This activates the unfolded protein response (UPR), a protective mechanism designed to mitigate damage from misfolded proteins. However, in severe mutations, such as those in *COL2A1*, the UPR may be insufficient, resulting in apoptosis (programmed cell death), particularly in chondrocytes (cells that form cartilage). This contributes to the disruption of cartilage and bone development.

B. Cartilage development is critical during endochondral ossification, where cartilage serves as a precursor to bone. In ACG 2, the mutated *COL2A1* gene leads to defective cartilage matrix formation, especially in the growth plate cartilage. As a result, cartilage formation and maintenance are impaired, severely affecting bone growth in the limbs and ribs. This results in short, deformed bones (micromelia) and a narrow chest due to underdeveloped ribs. The failure of the growth plates in the long bones of the spine to ossify properly leads to profound skeletal abnormalities.

The above two mechanisms are not mutually exclusive. Both mechanisms occur simultaneously, and they are interconnected parts of the same pathogenic cascade. Type II collagen is the essential structural protein for cartilage. A mutation that causes misfolding (mechanism A) will inevitably reduce functional collagen secretion, damage chondrocytes through ER stress, and lead to the collapse of cartilage development (mechanism B).

Therefore, mechanism B cannot occur without mechanism A, and mechanism A has no clinical significance without mechanism B. So, both mechanisms co-occur and are mechanistically linked. Mechanism A is the molecular defect, but mechanism B is the anatomic consequence.

The most prominent features of ACG 2 are short, malformed limbs and an underdeveloped rib cage. These abnormalities result from defects in cartilage formation and rib ossification, leading to a small, narrow thorax. This severely compromises respiratory function, which is the leading cause of death in affected infants. Micromelia, characterized by shortened limbs, is a hallmark of the disorder and reflects the arrested development of long bone growth due to impaired cartilage formation.

Prenatal imaging demonstrated micrognathia with a flattened facial profile. Although quantitative data on its incidence are lacking, micrognathia is consistently described as a characteristic prenatal ultrasound feature of achondrogenesis, often accompanied by a flat or hypoplastic midface as part of the typical facial dysmorphism in affected fetuses; however, in our clinical practice, it is rare [31].

The prognosis for individuals with ACG 2 is very poor [32]. Most affected infants do not survive long after birth due to respiratory failure caused by the underdeveloped rib cage and lungs. Some may survive for a few days to weeks, but severe skeletal deformities typically result in fatal complications during early infancy.

In conclusion, the pathogenesis of ACG 2, also known as Achondrogenesis Type II, resulting from missense mutations in the *COL2A1* gene, involves a complex interplay of defective collagen production, impaired cartilage formation, and cellular stress. These processes severely disrupt skeletal development, leading to fatal outcomes [33,34,35].

In our study, high-resolution advanced ultrasound initially revealed abnormal findings in this fetus: prominent skin edema and shortening of the femur, while the biparietal diameter (BPD) and abdominal circumference (AC) were within normal ranges for gestational age. Upon further examination of all the long bones, we observed marked micromelia, with nearly all measurements falling below the 1st percentile (except for radial length, which was below the 3rd percentile). Additionally, we noted the absence of ossification in the vertebral bodies, with ossification only apparent in the bilateral vertebral pedicles. This was evident in both the cross-sectional and coronal planes using 2D and 3D ultrasound (Figure 1 and Figure 2). One advantage of 3D ultrasound was its ability to display three sections simultaneously, particularly the coronal plane of the fetal spine (Figure 2C). This view helps confirm the absence of vertebral body ossification, which is crucial for differentiating ACG 2. Another advantage of 3D ultrasound (Figure 2A) is that it can provide additional confirmation from a side view when highly suspected micrognathia and a flat, hypoplastic midface are initially seen on 2D ultrasound.

## 6. Conclusions

Mutations in the *COL2A1* gene, responsible for Achondrogenesis Type II (ACG II), are primarily missense mutations, with glycine substitutions being the most common. These mutations highlight the crucial role of glycine in maintaining the structural integrity and function of collagen. The case presented in this report involves a glycine-to-serine substitution at amino acid position 516 in the *COL2A1* gene.

Due to this congenital abnormality, it is recommended that the woman and her partner undergo genetic counseling in any future pregnancies.

## Data Availability

The datasets used in the current study are available from the corresponding author upon reasonable request.

## Supplementary Materials

The following supporting information can be downloaded at: https://www.mdpi.com/article/10.3390/ijms262311472/s1.
