# Supplementary material for: Prenatal Imaging of Micrognathia, Micromelia, and Fetal Hydrops Leading to the Diagnosis of Achondrogenesis Type II with a COL2A1 Missense Mutation
Source: Int J Mol Sci. 2025 Nov 27;26(23):11472. doi: 10.3390/ijms262311472 (PMC12692501; doi:10.3390/ijms262311472)
Supplement: Supplementary file 1 [file ijms-26-11472-s001.zip › Supplementary Materials Table S1.pdf]

Supplementary Materials:

Table S1 Nucleotide substitution and missense mutation in human COL2A1 that causes Achondrogenesis II

No.	Location of the exon	Protein Change	cDNA variant (NM_001844.5)	Mutation Type	Mutation Effect	Phenotype	Reference	PMID
**1**	Exon 6	p.Arg137Cys	c.409C>T	Substitution	Missense	ACG 2	Barat-Houari 2016	PMID: 26626311
**2**	Exon 11	p.Gly253Val	c.757G>A	Substitution	Missense	ACG 2	Korkko 2000	PMID: 10797431
**3**	Exon 11	p.Gly253Asp	c.757G>A	Substitution	Missense	ACG 2	Korkko 2000	PMID: 10797431
**4**	Exon 15	p.Gly310Asp	c.928G>A	Substitution	Missense	ACG 2	Bonaventure 1995	PMID: 7741714
**5**	Exon 15	p.Gly313Ser	c.937G>A	Substitution	Missense	ACG 2	Mortier 2000	PMID: 10745044
**6**	Exon 17	p.Gly346Val	c.1037G>T	Substitution	Missense	ACG 2	Forzano 2007	PMID: 17994563
**7**	Exon 19	p.Gly391Ala	c.1171G>C	Substitution	Missense	ACG 2	Mortier 2000	PMID: 10745044
**8**	Exon 19	p.Gly402Glu	c.1205G>A	Substitution	Missense	ACG 2	Nishimura 2005	PMID: 15895462
**9**	Exon 21	p.Gly426Arg	c.1276G>C	Substitution	Missense	ACG 2	Nishimura 2005	PMID: 15895462
**10**	Exon 21	p.Gly447Asp	c.1340G>A	Substitution	Missense	ACG 2	FAIVRE 2004	PMID: 15054848
**11**	Exon 21	p.Gly447Asp	c.1340G>A	Substitution	Missense	ACG 2	Faivre 2004	PMID: 15054848
**12**	Exon 21	p.Gly450Asp	c.1349G>A	Substitution	Missense	ACG 2	Barat-Houari 2016	PMID: 26626311
**13**	Exon 21	p.Gly453Ser	c.1357G>A	Substitution	Missense	ACG 2	Nishimura 2005	PMID: 15895462
**14**	Exon 22	p.Gly459Asp	c.1376G>A	Substitution	Missense	ACG 2	Nishimura 2005	PMID: 15895462
**15**	Exon 22	p.Gly468Val	c.1403G>T	Substitution	Missense	ACG 2	Nishimura 2005	PMID: 15895462
**16**	Exon 24	p.Gly510Asp	c.1529G>A	Substitution	Missense	ACG 2	Heinrich 2015	PMID: 25823796
**17**	Exon 25	p.Gly517Val	c.1594G>T	Substitution	Missense	ACG 2	Mortier 2000	PMID: 10745044
**18**	Exon 26	p.Gly571Asp	c.1711G>A	Substitution	Missense	ACG 2	Korkko 2000	PMID: 10797431
**19**	Exon 26	p.Gly574Ser	c.1720G>A	Substitution	Missense	ACG 2	Horton 1992	PMID:1374906
**20**	Exon 27	p.Gly591Asp	c.1772G>A	Substitution	Missense	ACG 2	Barat-Houari 2016	PMID: 26626311
**21**	Exon 27	p.Gly595Arg	c.1784G>A	Substitution	Missense	ACG 2	Korkko 2000	PMID: 10797431
**22**	Exon 27	p.Gly604Ala	c.1811G>C	Substitution	Missense	ACG 2	Freisinger 1994	PMID: 8175802
**23**	Exon 32	p.Gly691Arg	c.2071G>C	Substitution	Missense	ACG 2	Mortier 1995	PMID: 7757081
**24**	Exon 32	p.Gly691Arg	c.2071G>C	Substitution	Missense	ACG 2	Williams 1995	PMID: 7757086
**25**	Exon 32	p.Gly694Glu	c.2080G>A	Substitution	Missense	ACG 2	Korkko 2000	PMID: 10797431
**26**	Exon 34	p.Gly748Asp	c.2242G>T	Substitution	Missense	ACG 2	Korkko 2000	PMID: 10797431
**27**	Exon 35	p.Gly768Asp	c.2303G>A	Substitution	Missense	ACG 2	Comstock 2010	PMID: 20583175
**28**	Exon 35	p.Gly768Asp	c.2303G>A	Substitution	Missense	ACG 2	Comstock 2010	PMID: 20583175
**29**	Exon 35	p.Gly769Ser	c.2305G>A	Substitution	Missense	ACG 2	Chan 1995	PMID: 7829510
**30**	Exon 35	p.Gly781Ser	c.2341G>A	Substitution	Missense	ACG 2	Korkko 2000	PMID: 10797431

**31**	Exon 36	p.Gly795Arg	c.2383G>C	Substitution	Missense	ACG 2	Korkko 2000	PMID: 10797431
**32**	Exon 37	p.Gly805Ser	c.2413G>A	Substitution	Missense	ACG 2	Bonaventure 1995	PMID: 7741714
**33**	Exon 38	p.Gly822Asp	c.2465G>A	Substitution	Missense	ACG 2	Barat-Houari 2016	PMID: 26626311
**34**	Exon 39	p.Gly853Glu	c.2557G>A	Substitution	Missense	ACG 2	Bogaert 1992	PMID: 1429602
**35**	Exon 39	p.Gly865Val	c.2593G>A	Substitution	Missense	ACG 2	Korkko 2000	PMID: 10797431
**36**	Exon 41	p.Gly910Cys	c.2728G>T	Substitution	Missense	ACG 2	Mortier 2000	PMID: 10745044
**37**	Exon 42	p.Gly919Arg	c.2755G>T	Substitution	Missense	ACG 2	Korkko 2000	PMID: 10797431
**38**	Exon 42	p.Gly936Asp	c.2807G>A	Substitution	Missense	ACG 2	Nishimura 2005	PMID: 15895462
**39**	Exon 42	p.Gly943Ser	c.2827G>A	Substitution	Missense	ACG 2	Mortier 2000	PMID: 10745044
**40**	Exon 43	p.Gly988Arg	c.2962G>C	Substitution	Missense	ACG 2	Williams 1995	PMID: 7757086
**41**	Exon 47	p.Gly1095Cys	c.3285G>T	Substitution	Missense	ACG 2	Mundlos 1996	PMID: 8723098
**42**	Exon 48	p.Gly1143Ser	c.3427G>A	Substitution	Missense	ACG 2	Vissing 1989	PMID: 2572591
**43**	Exon 50	p.Gly1188Arg	c.3563G>C	Substitution	Missense	ACG 2	Nishimura 2005	PMID: 15895462
**44**	Exon 50	p.Thr1191Asn	c.3571C>A	Substitution	Missense	ACG 2	Mortier 2000	PMID: 10745044
**45**	Exon 50	p.Gly1200Ser	c.3598G>A	Substitution	Missense	ACG 2	Nishimura 2005	PMID: 15895462
**46**	Exon 51	p.Gly1200Cys	c.3598G>T	Substitution	Missense	ACG 2	Nishimura 2005	PMID: 15895462
**47**	Exon 53	p.Gly1405Ser	c.4213G>A	Substitution	Missense	ACG 2	Vissing 1989	PMID: 2572591
**48**	Exon 53	p.Gly1405Ser	c.4213G>A	Substitution	Missense	ACG 2	Vissing 1989	PMID: 2572591
**49**	Exon 53	p.Gly1405Ser	c.4213G>A	Substitution	Missense	ACG 2	Vissing 1989	PMID: 2572591
**50**	Exon 53	p.Gly1405Ser	c.4213G>A	Substitution	Missense	ACG 2	Vissing 1989	PMID: 2572591
This case.	Exon 24	p.Gly516Ser	c.1703G>A	Substitution	Missense	ACG 2		

(This Table originated from Supplementary Table S1 in Clin Genet.2020;97:383-395)
