# Supplementary material for: Prenatal Imaging of Micrognathia, Micromelia, and Fetal Hydrops Leading to the Diagnosis of Achondrogenesis Type II with a COL2A1 Missense Mutation
Source: Int J Mol Sci. 2025 Nov 27;26(23):11472. doi: 10.3390/ijms262311472 (PMC12692501; doi:10.3390/ijms262311472)
Supplement: Supplementary file 1 [file ijms-26-11472-s001.zip › Supplementary S1.pdf]

Major Differences Between A and B

1. Transcript Version (NM number)

A: “c.1546G>A with NM_001844.5”

- Uses the current HGVS standard.
- Annotation is based on the latest transcript version: NM_001844.5.
- This is the updated reference sequence and may include corrected exon boundaries, UTR adjustments, or coding-region shifts.

B: “1703G>A with NM_001844 (no version specified)”

- Uses an older transcript version (often NM_001844.1 when no version is specified).
- Earlier GenBank records may differ from NM_001844.5 in coding sequence length or exon numbering.

Conclusion:
A and B are based on different transcript versions, so the cDNA positions 1546 and 1703 do not refer to the same nucleotide.

---

2. cDNA Position vs HGVS Update

A: c.1546G>A

- The variant is mapped to c.1546 according to the latest HGVS rules and updated reference sequence NM_001844.5.
- This is the correct modern HGVS notation.

B: 1703G>A

- Based on a previous annotation, before HGVS updated the numbering based on the revised transcript.
- The exact genomic change can shift position when transcript versions are updated.

Conclusion:
1546G>A and 1703G>A are the *same genomic variant*, but renumbered due to transcript updates.

---

3. Protein Description

A

(1) Only describes the DNA change, no protein effect given.

(2) But by using NM_001844.5, it would translate to p.Gly516Ser.

B

(1) Explicitly states the protein change:
p.Gly516Ser (NP_001835).
(2) Correctly describes glycine → serine at amino acid position 516.

Conclusion:
Both A and B refer to the exact amino acid change (Gly516Ser), but only B states it explicitly.

---

4. Modernity & Accuracy

A is the correct, current HGVS-compliant version

- Uses NM_001844.5.
- Uses updated cDNA position (1546).
- Is the preferred form for clinical genetics, ACMG classification, and publications.

B is outdated but historically used

- Uses an older transcript.
- Uses the older cDNA position (1703).
- Still describes the same amino acid substitution.

Summary Table

Feature	A: c.1546G>A (NM_001844.5)	B: 1703G>A (old NM_001844)
Transcript version	NM_001844.5 (current)	Older transcript
cDNA numbering	1546	1703
Reason for the difference	Updated transcript annotation changes positions	Older annotation
Protein effect listed?	Not shown, but is p.Gly516Ser	Explicit p.Gly516Ser
HGVS compliance	Yes (correct)	No (outdated)
Are they describing the same variant?	Yes	Yes

Final Answer:

A and B describe the same underlying genomic variant (COL2A1 p.Gly516Ser). Still, the cDNA numbering is different because A uses the updated HGVS-compliant transcript NM_001844.5 (c.1546G>A). In contrast, B uses an older transcript version that numbered the same nucleotide as c.1703G>A.
