# Supplementary figures and images for: Prenatal Imaging of Micrognathia, Micromelia, and Fetal Hydrops Leading to the Diagnosis of Achondrogenesis Type II with a COL2A1 Missense Mutation
Source: Int J Mol Sci. 2025 Nov 27;26(23):11472. doi: 10.3390/ijms262311472 (PMC12692501; doi:10.3390/ijms262311472)

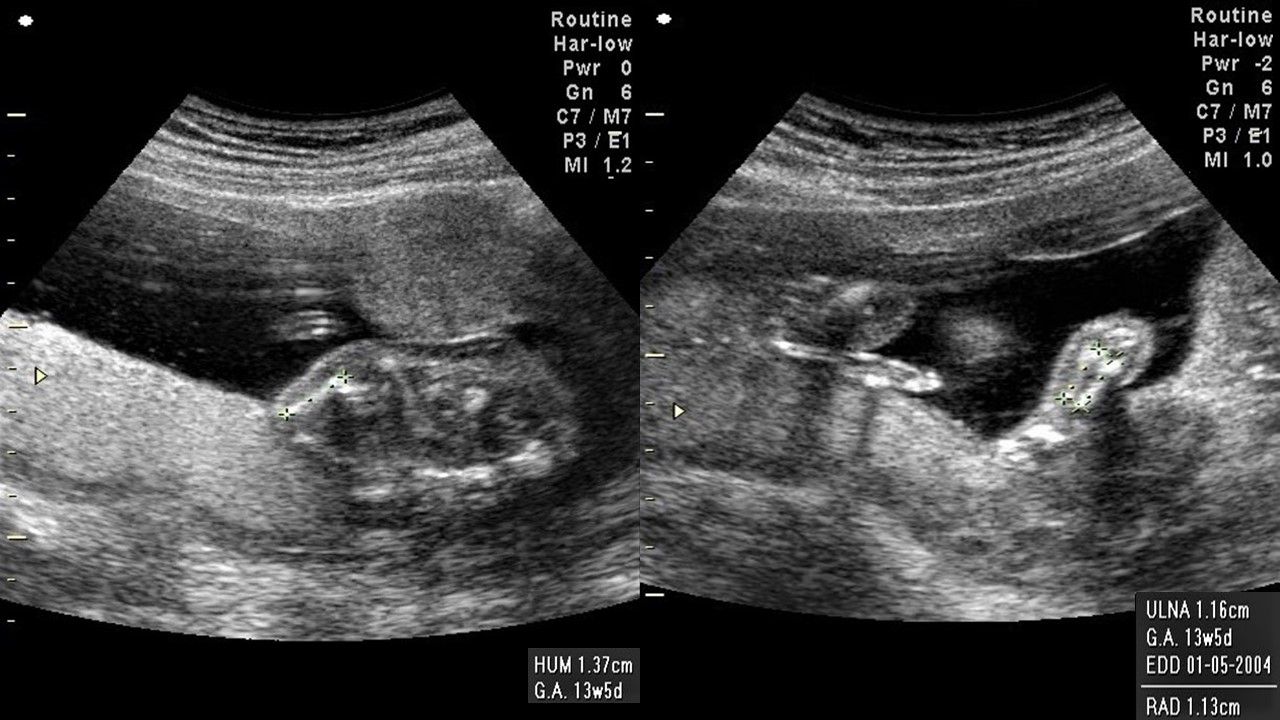

Supplement: Supplementary file 1 [file ijms-26-11472-s001.zip › Figure A.JPG]

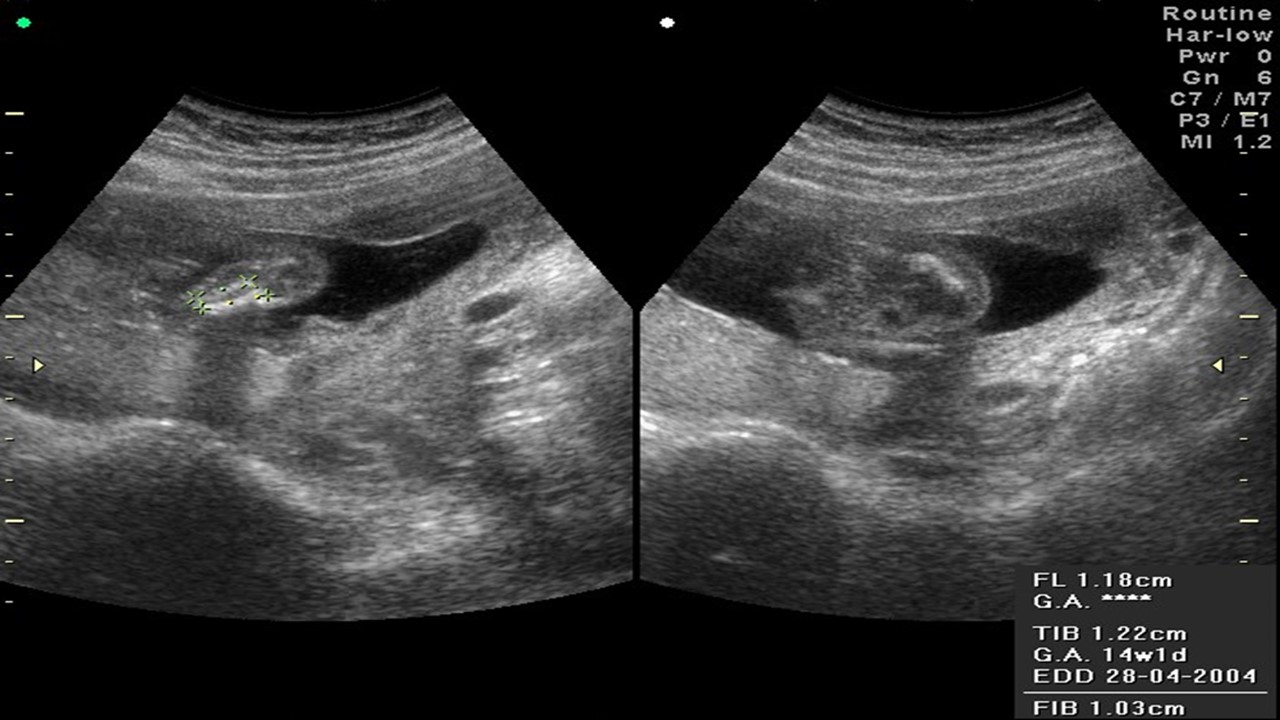

Supplement: Supplementary file 1 [file ijms-26-11472-s001.zip › Figure B.JPG]
